# Unsolved Controversies in Management of Infective Endocarditis

**DOI:** 10.7759/cureus.58095

**Published:** 2024-04-12

**Authors:** Rima Othman, Hassan H Mallat, Raed Osman, Ahmad Ayoubi

**Affiliations:** 1 General Medicine, Faculty of Medicine at University of Balamand, Beirut, LBN; 2 Infectious Diseases, Doctoral School of Sciences and Technology, Faculty of Public Health, Lebanese University, Tripoli, LBN; 3 Interventional Cardiology, Nini Hospital, Tripoli, LBN; 4 Cardiac Surgery, Nini Hospital, Tripoli, LBN

**Keywords:** mitral chordae rupture, s aureus, pediatric infection, mitral valve surgery, infective endocarditis

## Abstract

Infective endocarditis (IE) is defined as an infection in the cardiac endothelium. It is triggered by both bacteremia and endothelial dysfunction and poses many risks to the health of the patient. Many organisms can cause IE with *Staphylococcus aureus* being the major cause. Signs and symptoms may vary according to age and agent but almost all cases are presented with fever, fatigue, and a maculopapular rash. Although pediatric IE is very rare, risk factors such as congenital heart defects have been identified, with some of the cases remaining a mystery. We present a case of a 19-year-old patient, previously healthy and developing subacute IE with sepsis and septic embolic showers in multiple organs. IE cannot be taken for granted as mortality is high, hence a multidisciplinary approach is prompt and necessary for the survival of the patient.

## Introduction

Infective endocarditis (IE) is the infection of the heart endothelium. It is a rare condition affecting five out of 100,000 people per year, but it carries a high morbidity and mortality of 30% mortality in 30 days [1]. Many organisms can cause IE, with *Staphylococcus aureus* as the main culprit causing IE in almost 26% of cases as reported by the literature [1]. The clinical picture of IE can vary widely between patients (subacute, acute, or chronic), with presentation depending on the causative agent and patient-specific risk factors [2]. The pathogenesis of IE is widely attributed to a multi-hit theory implicating endothelial dysfunction and bacteremia as precursors [3]. In our case report, we will present the case of a 19-year-old previously healthy patient who was found to have IE, highlighting the nuances in the management of IE as tailored to our patient.

## Case presentation

**Figure 1:**
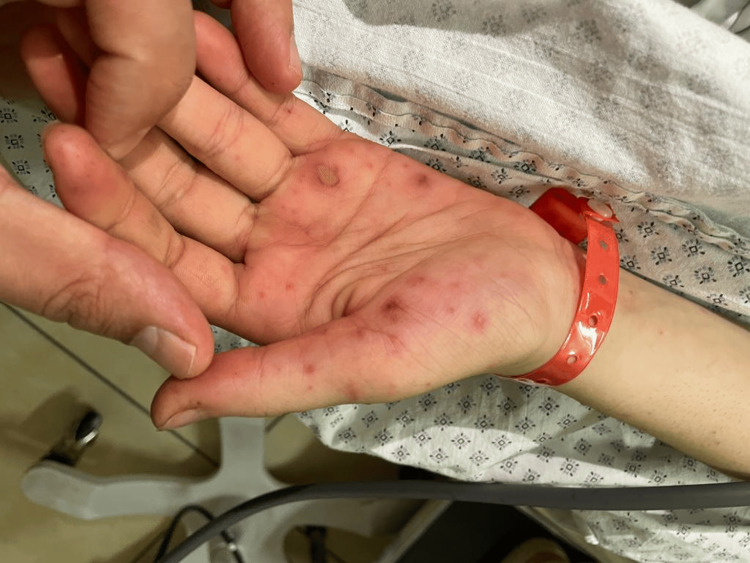
Osler nodes apparent on hands

**Figure 2:**
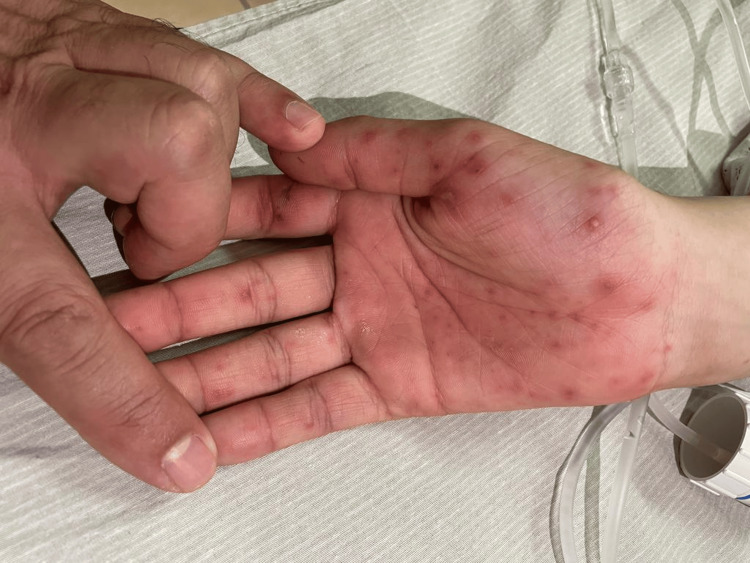
Osler nodes and Janeway lesions

This is the case of a 19-year-old previously healthy female patient, who presented to the emergency department of a peripheral hospital with a self-reported fever of four-day duration with a maculopapular rash on palms and soles (Figures 1, 2). Detailed history collected from the patient revealed no recent dental procedures. A recent episode of folliculitis in the scalp was noted. After physical examination, the patient was found to be in sinus tachycardia and hypotension and was accordingly resuscitated. The rash was identified as Osler nodes and Janeway lesions.

**Figure 3:**
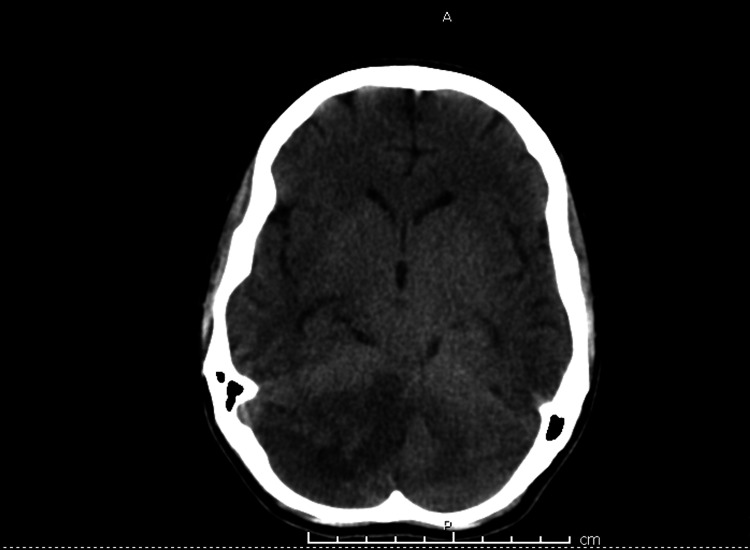
Brain CT showing cerebellar hemorrhage due to septic emboli

**Figure 4:**
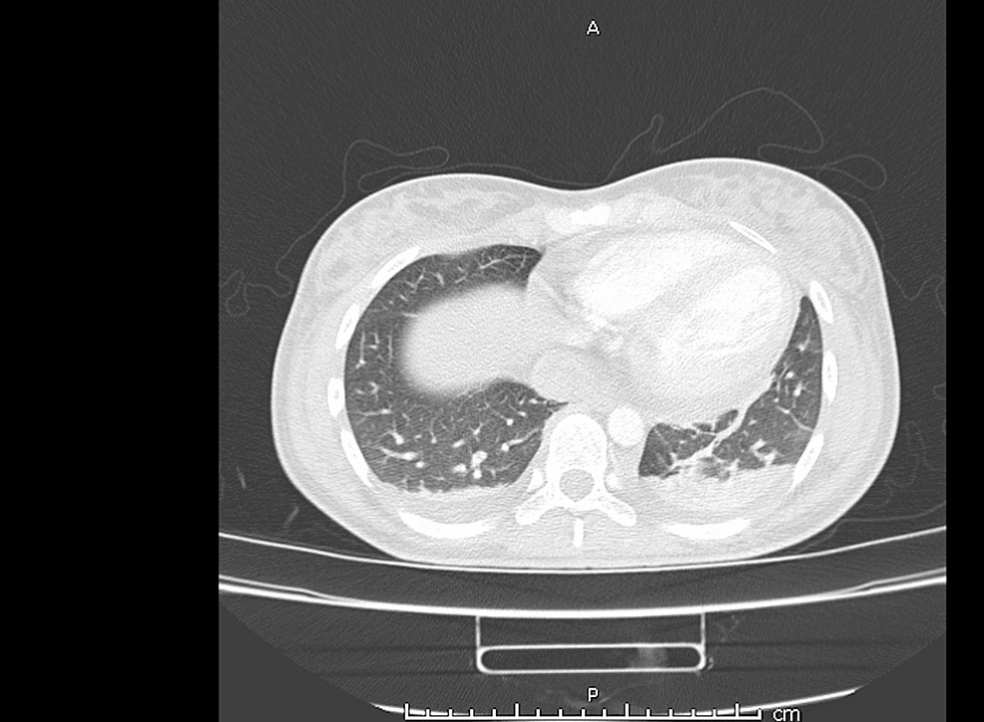
CT chest in the pulmonary window showing bilateral pleural effusion

**Figure 5:**
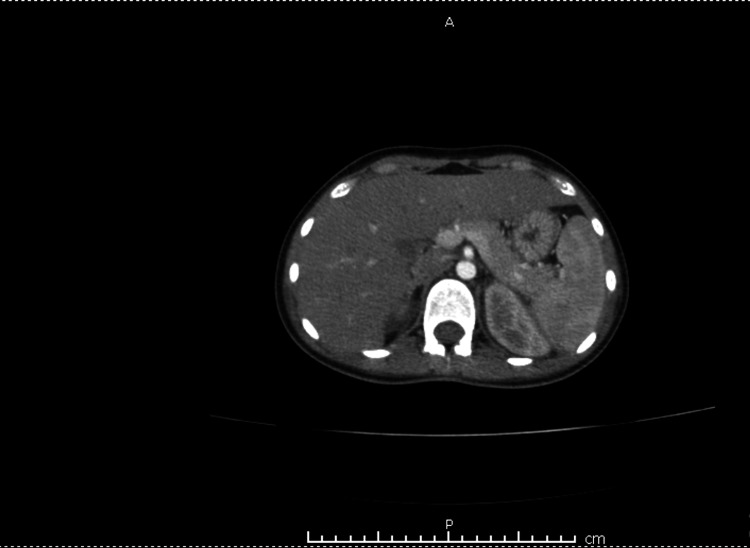
Abdominal CT showing hepatosplenomegaly with embolic showers

The patient was transferred to our center. Laboratory findings on presentation showed a CRP 295 (normal upper limit of our lab 50 mg/dL), troponin 139.3 ng/mL, and creatine kinase (CK) 196 U/L (normal upper limit of our lab 145 U/L). Upon preliminary evaluation using transthoracic echocardiogram, vegetations were observed on the mitral valve, and the patient was diagnosed with endocarditis and admitted to the hospital. The patient was started on ceftriaxone 2 mg BID, vancomycin 1 g BID, and gentamycin 80 mg BID in the peripheral hospital. On admission, repeat transthoracic echo cardio confirmed the preliminary diagnosis of infective endocarditis, hence medication regimen was unchanged. Detailed echocardiographic findings showed mitral valve 3 cm annular dilatation, vegetations on the posterior mitral leaflet, and mild pericardial effusion. Pan cultures were taken, and a full body CT was ordered. CT showed septic emboli in the brain specifically, the right occipital lobe (Figure 3), bilateral pleural effusions (Figure 4), bilateral basal lung collapse, hepatosplenomegaly with diffuse ischemia and superior mesenteric ischemia (Figure 5).

**Figure 6:**
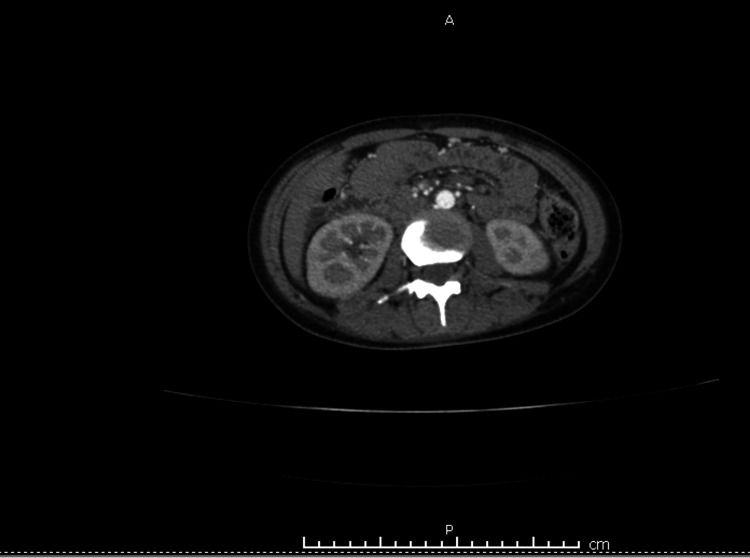
Abdominal CT showing kidney infarcts due to septic emboli

Microinfarcts were also noted in kidneys and pelvic ascites were present (Figure 6). Brain MRI was sequentially ordered for further investigation, and results revealed ischemic microinfarcts in the right cerebellum as well as the right occipital lobe with no evidence of hemorrhage.

Cultures taken on admission grew methicillin-resistant *Staphylococcus aureus *(MRSA), and antibiotics were changed according to the obtained antibiogram. The patient remained febrile with no afebrile window lasting more than 24 hours throughout the entire window of diagnostic testing. Vancomycin was increased to 2 g Q24h as patient inflammatory markers were not trending down as expected. Gentamycin was stopped on day 11, and one packed red blood cell (pRBC) was transfused due to a hemoglobin (Hb) drop of 1.5 units. Ciprofloxacin 400 mg BID was added on day 13 when a repeat chest X-ray showed signs of bilateral pulmonary infiltrates. On day 21 after admission (day 30 or reported symptoms), the patient developed flash pulmonary edema with a new onset murmur which was not present on admission, and the surgical team was accordingly consulted. Guidelines specify that the benefits of cardiac surgery are more prominent after one month of onset of the disease, but we were hesitant to progress into the surgical approach with cerebral emboli present. Our patient had relapsed exactly 30 days after symptom onset and fulfilled surgical criteria, thus we proceeded with mitral valve replacement. The operation was successful and patient symptoms and studies improved. The patient was discharged on vancomycin 500 mg TID and linezolid 600 mg BID as well as Sintrom 4 mg once daily one week after the procedure. Vancomycin was tapered to 500 mg TID prior to discharge based on vancomycin levels of 45 mg. The patient was counseled on proper hygiene and anticoagulation management with follow-up as an outpatient treatment.

## Discussion

Traditionally, the diagnosis of IE is done using the Duke’s criteria or modified Duke’s criteria and antibiotics should not be administered until 3 sets of blood cultures confirm the state of bacteremia with high suspicion of cardiac origin. The updated Duke's criteria does not focus on the number of positive cultures but on the certainty of diagnosis [4]. In our case, the high clinical suspicion of IE as well as the hemodynamic instability prompted rapid action and administration of antibiotics immediately after cultures were taken [1]. The high suspicion of IE in this case was based on our patient's age group, socioeconomic status, and presentation, despite no previous history of valvulopathy or systemic infection [5].

Our patient had an atypical presentation as compared to what is usually reported in IE cases. based on the obtained history, the only possible episode that could have caused the IE was scalp folliculitis. In cases of endocarditis, systemic infections are commonly the trigger, as opposed to what we observed. Moreover, patients usually have a history of valve disease, valve replacement, or prior endocarditis. Our patient possessed a previously healthy, native valve with no history of injury or childhood infections. 

In our patients' age group, clear guidelines remain rather limited with the majority of available literature detailing management in older adults with prosthetic devices or indwelling catheters posing as a clear focus of infection [5,6]. The lack of set guidelines for the management of severe IE in adults posed a challenge, prompting us to tailor traditional guidelines as the best fit for our patient. Clinical trials are traditionally considered the preferred scientific resource for clinical management, however, we find it highly unique that a clinical trial for the management of IE in healthy young adults with no prior history will take place anytime in the near future due to the rarity of such cases.

For our patient, frequent imaging modalities and biomarkers were considered as sensors for the response to management [7]. A transthoracic echocardiogram (TTE) and control CT scans were done once weekly, as well as daily labs to follow up on trends in biomarkers. The discrepancy between imaging and intra-operative findings was minimal, despite some studies suggesting that significant differences may be found. In our case, the patient was only found to have an abscess near the posterior mitral leaflet as expected clinically prior to surgery. This was considered a positive finding, as it implied that the management course was proper and fitting to the patient. Multiple controversies are present in the field of IE management. Following our reported course of management, our young, previously healthy patient had a comparable response to that of older individual with prior comorbidities. we suggest similar regimens may play a key role in the prognosis of MRSA induced IE in previously healthy young adults.

## Conclusions

After presenting our case and the literature behind it, we highlight the rarity of infective endocarditis in a young, healthy patient after only one episode of folliculitis. The literature is sparse in this age group and hence triggers a question of whether new guidelines for diagnosis and treatment should be set forth. Patients in this category may have different triggers and presentations, as seen with our patient who had only one prior, minor infection of the scalp, and thus may require different criteria for diagnosis and subsequent treatment. 
